# Circulating MicroRNA-4739 May Be a Potential Biomarker of Critical Limb Ischemia in Patients with Diabetes

**DOI:** 10.1155/2018/4232794

**Published:** 2018-11-14

**Authors:** Ju-yi Li, Biao Cheng, Xiu-fang Wang, Zhong-jing Wang, Hong-mei Zhang, Shu-fen Liu, Li-sha Chen, Wen-jing Huang, Jue Liu, Ai-ping Deng

**Affiliations:** ^1^Department of Pharmacy, The Central Hospital of Wuhan, Tongji Medical College, Huazhong University of Science and Technology, Wuhan 430021, Hubei, China; ^2^Department of Pain, The Central Hospital of Wuhan, Tongji Medical College, Huazhong University of Science and Technology, Wuhan 430021, Hubei, China; ^3^Department of Endocrinology, The Central Hospital of Wuhan, Tongji Medical College, Huazhong University of Science and Technology, Wuhan 430021, Hubei, China

## Abstract

Critical limb ischemia (CLI) is the most severe manifestation of peripheral artery disease, which is common but rarely diagnosed. Noninvasive biomarkers are urgently required to assist in the diagnosis of CLI. Accumulating evidence indicates that miRNAs play an important role in the development of various diseases. In this study, microarray profiling revealed 11 miRNAs with significantly altered expression in four T2DM patients with CLI compared with that in four sex- and age-matched T2DM patients without CLI. In independent cohorts, qRT-PCR validation confirmed the increased miRNA-4739 level in patients with CLI versus patients without CLI. miRNA-4739 levels increased with FPG and HbA1c (all* P *< 0.05). After adjusting for the risk factors, miRNA-4739 levels were found to be associated with an increased odds ratio (OR) of T2DM with CLI (OR =12.818, 95% confidence intervals (CI) 1.148 to 143.143,* P* = 0.038). ROC curve analysis revealed that the area under the curve (AUC) of miR-4739+confounding risk factors was 0.94 (95% CI 0.891 to 0.998,* P* < 0.001), which was higher than that of confounding risk factors (AUC 0.94* vs*. 0.91, 95% CI -0.122 to 0.060,* P* > 0.05) and of miR-4739 (AUC 0.94* vs*. 0.69, 95% CI -0.399 to -0.101,* P* < 0.001), respectively. We conclude that elevated plasma miRNA-4739 levels are independently associated with CLI in T2DM patients. miRNA-4739 is implicated as a novel diagnostic marker and a potential therapeutic target for CLI in diabetes.

## 1. Introduction

Type 2 diabetes mellitus (T2DM) is associated with accelerated peripheral arterial disease (PAD), which is characterized by the narrowing or occlusion of systemic arteries. This condition impeded blood supply to the extremities and is associated with significant cardiovascular morbidity and mortality [1, 2]. Critical limb ischemia (CLI) is the most severe manifestation of PAD and seriously decreases blood flow to the extremities, which increases the risk of foot ulcers, amputation, and vascular death, especially in diabetic patients [3].

The increasing number of people being diagnosed with diabetes mellitus, and the global escalation in the aging population, is expected to exacerbate the CLI burden [2]. T2DM is a major contributor to CLI and although the biological mechanisms remain to be fully elucidated, it is generally believed that contributing factors include oxidative and glycemic stress, chronic low grade inflammation, and impaired vascular tissue repair [4–6]. CLI therapy includes statins, antiplatelet therapy, revascularization, and even amputation to reduce cardiovascular risk factors, morbidity, and mortality [7, 8]. Currently, the prognosis of patients with CLI is very poor. Only 10% to 30% of patients with PAD have the classic clinical symptoms [9]; however, patients with a confirmed diagnosis of CLI have a 25% to 40% chance of lower limb amputation and approximately a 20% chance of mortality, with severely diminished quality of life [2, 10].

Currently, the ankle-brachial index (ABI), color Doppler ultrasound scans, and transcutaneous oxygen pressure are used to confirm the diagnosis of CLI [11]. Previous studies have implicated some serum or plasma proteins, including Siglec-5, cystatin C, haptoglobin, and 14-3-3 protein zeta as potential biomarkers of CLI in diabetes [3, 12, 13]; however, these markers have not been confirmed due to the limited accuracy of the currently available detection technologies. Therefore, specific biomarkers are urgently required as early diagnostic markers of CLI in T2DM patients to reduce the high incidence of amputations.

MicroRNAs (miRNAs) are small (18–22 nucleotides) noncoding RNA molecules that regulate the expression of hundreds of mRNAs at the posttranscriptional level through binding to target mRNA, resulting in either mRNA degradation or translational repression [14, 15]. miRNAs are present in extracellular spaces and affect many physiological processes [16]. Studies suggest that circulating microRNA levels are associated with angiogenesis, endothelial function, and circulating proangiogenic cell functions in patients with CLI [17–20]. miRNAs are remarkably stable in the circulation and are useful as biomarkers or diagnostic tools for a number of diseases [21, 22].

In this study, we analyzed the expression levels of microRNAs in plasma obtained from patients with T2DM (4 with CLI and 4 patients without CLI matched for age, sex, and diabetes duration) using a microRNA array containing all human miRNAs. The candidate microRNAs identified in independent cohorts were validated by quantitative reverse transcription-polymerase chain reaction (qRT-PCR). Thus, in this study, we aimed to identify a novel diagnostic biomarker of CLI in patients with a confirmed T2DM diagnosis by screening plasma microRNAs.

## 2. Materials and Methods

### 2.1. Subjects

This study was approved by the Ethics Committee of the Central Hospital of Wuhan (China) and all participants provided written informed consent. Patients diagnosed with T2DM were recruited from the Central Hospital of Wuhan from May 2015 to May 2017. T2DM was diagnosed according to the current American Diabetes Association standards [23].

Following TASC 2007 criteria CLI was defined as pain at rest, and/or ulcer or gangrene due to peripheral artery disease, transcutaneous oximetry at the dorsum of the foot, 30 mmHg, and/or ankle pressure, 70 mmHg, ABI <0.9, and lower extremity arterial stenosis >50% shown in color Doppler ultrasound scans. Patients with ABI ≥0.9 and <1.3 and normal lower extremity arterial pressure were defined as non-CLI [3, 12]. For the initial biomarker discovery studies using protein arrays, non-CLI controls were appropriately matched for sex, age (±5 years), and T2DM duration (±5 years) with the group of patients with CLI. According to these criteria, the patients with T2DM recruited into this study were divided into two groups of participants with and without CLI. The microarray study was conducted in four T2DM patients with CLI (2 males and 2 females) and four sex- and age-matched T2DM patients without CLI. The mean age of T2DM patients with CLI was 65.00 ± 3.46 years and 65.50 ± 3.11 years in T2DM patients without CLI. The mean diabetes duration of T2DM patients with CLI was 10.00 ± 2.83 years and 12.25 ± 3.59 years in T2DM patients without CLI. qRT-PCR studies were performed on an independent cohort of 26 T2DM patients with CLI and 30 T2DM patients without CLI. Exclusion criteria included type 1 diabetes mellitus, cancer, and severe liver and kidney failure, and being on therapy for any chronic inflammatory disease.

### 2.2. Information Collection

Clinical information including details of sex, age, diabetes duration, body mass index (BMI), blood pressure, smoking and drinking habits, blood glucose, glycosylated hemoglobin (HbA1c), lipoprotein lipid levels and previous history of hypertension, hyperlipidemia, and coronary artery disease (CAD) was obtained. Hypertension, hyperlipidemia, and CAD were defined as previously reported [3, 12].

### 2.3. RNA Isolation

Fasting blood samples (5 mL) were collected in EDTA-coated tubes. Total RNA was extracted from cells in the plasma using the miRNeasy serum/plasma kit (TIANGEN: catalog number DP503, China) according to the manufacturer's instructions. RNA quality was determined using an Agilent 2100 Bioanalyzer (Agilent Technologies, Santa Clara, CA, USA). RNA was quantified using a Nanodrop 2000 Spectrophotometer (Thermo Scientific, Wilmington, DE, SA).

### 2.4. miRNA Microarray Hybridization

RNA samples were obtained from T2DM patients with CLI (*n *= 4) and without CLI (*n *= 4) who were matched for age, sex, and diabetes duration. miRNA expression profiling was performed using the miRCURY™ LNA Array, which contains 2,085 human miRNAs. The total RNA isolated from each subject was labeled using the miRCURY™ Power Labeling kit (Exiqon, Inc., Vedbaek, Denmark) and hybridized to miRCURY™ Arrays according to the standard protocol. Hybridization images were scanned using an Axon GenePix 4000B microarray scanner (Molecular Devices, Sunnyvale, CA, USA). Scanned fluorescence data (data extraction, median normalization, and quality control) were processed using GenePix Pro 6.0 software (Molecular Devices).

### 2.5. Quantitative Reverse Transcription-Polymerase Chain Reaction (qRT-PCR)

The microarray data were validated by qRT-PCR studies on an independent cohort of 26 T2DM patients with CLI and 30 T2DM patients without CLI using SYBR Green (TIANGEN, China; catalog number FP411) according to a previously described method [24]. RNA was reverse-transcribed to cDNA using a reverse transcriptase kit (TIANGEN; catalog number: KR211) according to the manufacturer's instructions. miRNAs were then analyzed by qRT-PCR using the following conditions: 95°C for 1 min, followed by 40 cycles of 95°C for 10 s, 60°C for 30 s, and 70°C for 10 s. Duplicate assays were performed for each sample. The hsa-miR-93-5p value from the duplicate was used as the internal control [25]. The relative expression of each miRNA after normalization against hsa-miR-93-5p was calculated according to the following formula: 2 -^[Ct  (miRNA)  -  Ct  (hsa-miR-93-5p)]^.

### 2.6. Target Prediction and Functional Analysis

The gene targets of miR-4739 were predicted using two leading miRNA target prediction algorithms: TargetScan 7.0 (http://www.targetscan.org/) and miRanda V5 (http://mirdb.org/mirdb/). The online program MatchMiner (http://discover.nci.nih.gov/matchminer) was used to determine similar genes identified using these algorithms. Predicted miRNA targets were functionally annotated as biological process, cellular component, and molecular function by Gene Ontology (GO), and the pathways were described by the Kyoto Encyclopedia of Genes and Genomes (KEGG) database.

### 2.7. Statistical Analysis

All statistical analyses were performed using SPSS 19.0 (SP, Chicago, IL, USA). Continuous variables were expressed as means ± standard error of the mean (SEM) or median (interquartile range), and categorical variables were expressed as numbers and percentages. Fisher's exact test or the *x*^2^ test was used to compare categorical variables. An independent* t*-test or Fisher–Pitman Permutation test was used to compare normally distributed data, while a nonparametric Mann–Whitney* U* test or Exact Mann–Whitney rank sum test was used to compare non-normally distributed data.* P* values were calculated using the Benjamini and Hochberg method to correct for multiple-testing. Binary logistic regression analysis was performed to determine independent predictors of T2DM with CLI. All correlations were analyzed using the Pearson method or nonparametric Spearman method depending on the distribution (normal or nonnormal) of the data. Receiver operating characteristic curve (ROC) analysis was performed with plasma miR-4739 to distinguish between T2DM patients with and without CLI.* P* < 0.05 was considered to indicate statistical significance.

## 3. Results

### 3.1. Patient Characteristics

The main characteristics of the patients included in this study are summarized in Table 1. There were significant differences between the two groups of patients with and without CLI in terms of T2DM duration, hypertension, smoking, ABI, FPG, HbA1c, TC, and Cr (all* P *< 0.05), while there were no differences in the other parameters (all* P *> 0.05).

### 3.2. Identification of Differentially Expressed miRNAs in the Plasma of T2DM Patients with CLI and without CLI by Microarray Analysis

The microarray contained probes for 2,085 human miRNAs. After multiple-testing correction, six miRNAs (hsa-miR-589-5p, hsa-miR-3132, hsa-miR-4711-3p, hsa-miR-3935, hsa-miR-3168, and hsa-miR-4739) were shown to be significantly upregulated (mean ratio = 1.57–3.52,* P* < 0.05), while five miRNAs (hsa-miR-4434, hsa-miR-378i, hsa-miR-3135a, hsa-miR-514a-5p, and hsa-miR-378c) were significantly downregulated (mean ratio = 0.25–0.47,* P* < 0.05) in T2DM patients with CLI compared with those without CLI (Table 2).

**Figure 1:**
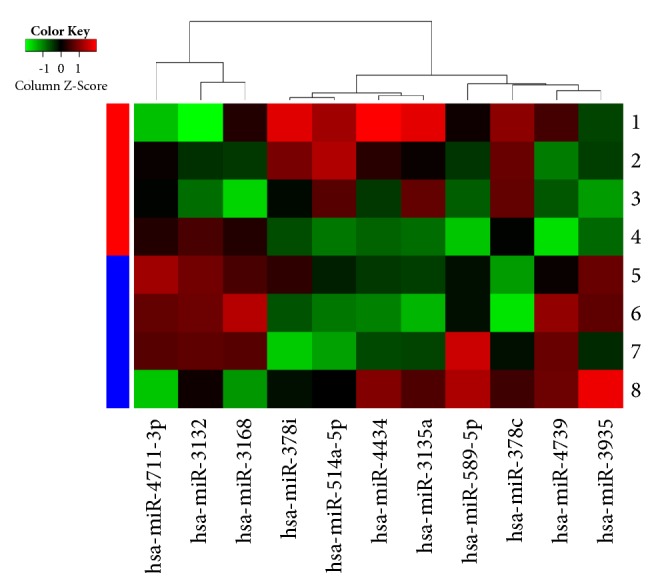
Heatmap of differentially expressed miRNAs between the T2DM with CLI group (1-4) and the T2DM without CLI controls (5-8).

A heatmap of the differentially expressed plasma miRNAs identified by comparison of T2DM patients with and without CLI was generated using GeneSpring software (Figure 1).

### 3.3. Validation of Microarray Data by Quantitative Real-Time PCR (qRT-PCR)

**Figure 2:**
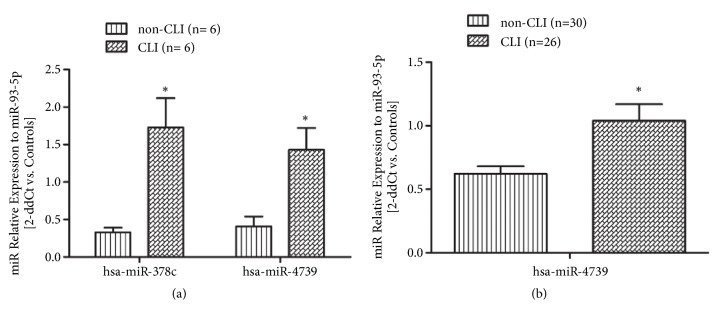
Validation of selected miRNAs in independent samples by qRT-PCR. (a) In cohort subjects T2DM with CLI (*n*= 6) and non-CLI (*n*= 6), level of hsa-miR-4739 is consistent with the initial discovery microarray study; however, hsa-miR-378c showed opposite direction of relative expression with the discovery microarray analysis. (b) Level of hsa-miR-4739 was further confirmed in an independent cohort 2 including T2DM with CLI (*n*= 26) and without CLI controls (*n*= 30) (*∗P*< 0.05).

Based on the level of regulation and their reported functional roles, and to rule out the possibility of false-positive results obtained in the microarray study, the expression patterns of the identified differentially expressed miRNAs (hsa-miR-4739 [upregulated] and hsa-miR-378c [downregulated]) were validated by qRT-PCR in an independent cohort 1 of T2DM patients with CLI (*n *= 6) and without CLI (*n *= 6). This analysis confirmed the significant difference in the expression level of hsa-miR-4739 between the T2DM patients with and without CLI. It is noteworthy that the relative expression of hsa-miR-378c determined by qRT-PCR in the validation cohort showed the opposite pattern to that determined in discovery microarray analysis, and it may be caused by the false positive results of abnormal high expression of individual samples in miRNA microarrays. The expression levels of hsa-miR-4739 were further confirmed in an independent cohort 2 of T2DM patients with CLI (*n *= 26) and without CLI (*n *= 30) (Figure 2).

### 3.4. Analysis of Plasma miRNA-4739 Level and CLI Risk Factors

Plasma miRNA-4739 levels increased with FPG and HbA1c, but decreased with the increase of ABI (all* P *< 0.05). There were positive associations between plasma miRNA-4739 levels and T2DM duration, hypertension, or smoking, and there were negative associations between plasma miRNA-4739 levels and TC or Cr, although these did not reach the level of statistical significance (all* P *> 0.05) (Table 3).

### 3.5. Risk Factors for CLI

Taking CLI as the dependent variable, the risk factors shown in Table 1 were entered into binary logistic regression analysis. The significant risk factors included T2DM duration, hypertension, smoking, FPG, HbA1c, TC, and Cr (Table 4). ABI was included in analysis and proved not to be significant (*P* = 0.993); therefore, this factor was excluded from our final model. After adjusting for the risk factors, plasma miRNA-4739 levels were found to be associated with an increased odds ratio (OR) of diabetes with CLI (OR = 12.818, 95% CI 1.148 to 143.143,* P* = 0.038) (Table 4).

### 3.6. The Diagnostic Value of miR-4739 for CLI

**Figure 3:**
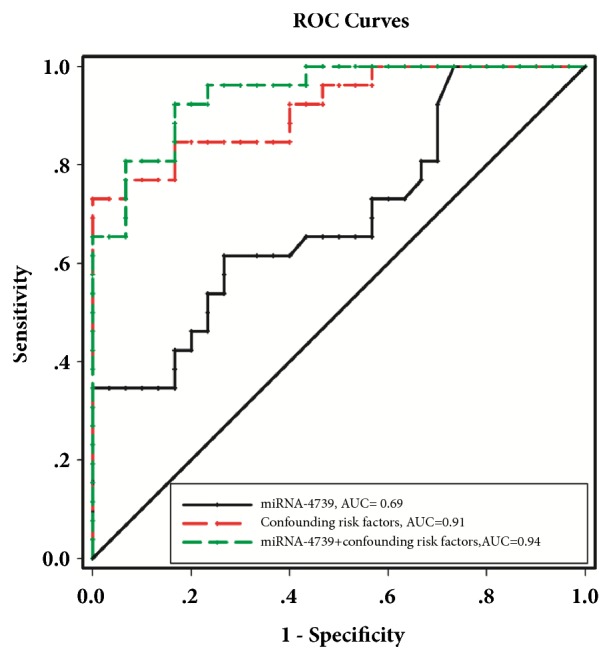
ROC curve analysis of miRNA-4739 cut point for the presence of CLI in T2DM patients. The AUC of miRNA-4739 was 0.69 (*P*< 0.05); identified miR-4739 cut point was 0.42, Youden index = 0.35; sensitivity: 61.54%; specificity: 73.33%. The AUC of confounding risk factors and miRNA-4739+ confounding risk factors were 0.91 and 0.94, respectively (*P*< 0.001).

ROC curve analyses revealed that miR-4739 levels represent a useful biomarker for the diagnosis of CLI in T2DM. The area under the curve (AUC) of miR-4739 and confounding risk factors was 0.69 (95% CI 0.556 to 0.834, P < 0.05) and 0.91 (95% CI 0.840 to 0.988, P < 0.001), respectively. The optimal cut-off point for miR-4739 was 0.42, at this level, the Youden index = 0.35, sensitivity was 61.54% (95% Cl 0.406 to 0.798), and specificity was 73.33% (95% Cl 0.541 to 0.877). Importantly, when the miR-4739 and confounding risk factors values were combined to form a composite panel, the resulting AUC was 0.94 (95% CI 0.891 to 0.998, P < 0.001), which was higher than that of confounding risk factors (AUC 0.94* vs*. 0.91, 95% CI -0.122 to 0.060,* P* > 0.05) and of miR-4739 (AUC 0.94* vs*. 0.69, 95% CI -0.399 to -0.101,* P* < 0.001), respectively. This indicated that the diagnostic performance of the miR-4739 and confounding risk factors panel was superior to either miR-4739 or confounding risk factors alone (Figure 3).

### 3.7. Target Prediction and Functional Analysis

In total, 251 similar target genes were identified using both the TargetScan 7.0 and miRDB algorithms; targets identified by only one of the prediction algorithms were excluded. In the analysis of the potential functions of these target genes using the KEGG pathways database, three biological pathways were identified (Table 5). GO enrichment analysis indicated that 41 GOs were significantly enriched by hsa-miR-4739, including regulation of transcription from the RNA polymerase II promoter, small GTPase-mediated signal transduction, and regionalization (data not shown).

## 4. Discussion

Altered levels of circulating miRNAs detected in body fluids, including blood, urine, and wound exudate, have been linked to various pathophysiological states [26]. A previous clinical study demonstrated that circulating miRNA-15a and miRNA-16 levels represent a prognostic biomarker in CLI patients undergoing revascularization treatment [19]. However, to date, regulation of plasma miRNAs in T2DM patients with CLI has not been reported. In our study, plasma hsa-miR-4739 levels were found to be significantly elevated in T2DM patients with CLI compared with those in T2DM patients without CLI. Hsa-miR-4739 was independently and strongly associated with T2DM patients with CLI, even after adjusting for risk factors (T2DM duration, hypertension, smoking, FPG, HbA1c, TC, and Cr). Therefore, our findings indicate that plasma hsa-miR-4739 levels represent a valuable diagnostic marker of T2DM patients with CLI, especially in T2DM patients without overt cerebrovascular disease [3].

Age, T2DM duration, smoking status, blood glucose, hyperlipidemia, and hypertension are the traditional risk factors for CLI [27]. In our study, we also found significant differences in T2DM duration, hypertension, smoking, FPG, and HbA1c between T2DM patients with and without CLI. However, plasma TC levels were lower in patients with CLI, possibly due to the poor nutritional status in this patient population. In addition, we detected higher plasma Cr levels in T2DM patients with CLI compared to those without CLI, which is consistent with previous reports [3, 12, 28]. In this study, this is the first time to report that plasma miRNA-4739 levels increased with FPG and HbA1c. This means that if blood glucose is poorly controlled, the plasma miRNA-4739 levels will increase significantly and increase the risk of critical limb ischemia.

MicroRNAs are relatively stable in serum or plasma and associated with membrane-bound vesicles or proteins. Due to the ease of sample handling and the availability of inexpensive qPCR-assays, microRNAs are ideal for use in clinical settings for screening of various diseases. Circulating miRNAs have been identified as potential noninvasive biomarkers for diagnosis and prognosis of multiple diseases [29]. Circulating levels of several miRNAs are modified by diabetes mellitus, and plasma miR-503 levels are increased in T2DM patients with CLI undergoing limb amputation [19, 30]. However, differential expression of other miRNAs in the plasma of T2DM patients with CLI patients has not yet been reported, which highlights the novelty and importance of our study. In the present study, we identified differentially expressed miRNAs in Chinese T2DM patients with CLI by combining microarray and qRT-PCR analyses. We found that plasma hsa-miR-4739 levels were significantly elevated in T2DM patients with CLI compared to those without CLI. Binary logistic regression analysis and ROC curve analysis revealed that hsa-miR-4739 was independently and strongly associated with T2DM patients with CLI and confirmed that hsa-miR-4739 plays an important role in diagnosis of CLI in T2DM patients.

Thus far, several studies have reported the functions of hsa-miR-4739, linking it to acute ischemic stroke, gastric cancer, acute myeloid leukemia, negative anaplastic large cell lymphoma, diabetic nephropathy, and osteogenic and adipocytic differentiation of immortalized human bone marrow stromal cells [31–33]. Little is known about the function of the miR-4739 and we identified only three signaling pathways including lysosomes by target prediction and GO analysis; therefore, we propose that that miR-4739 contributes to the development of CLI in patients with T2DM by modulating the autophagy-lysosome pathway; however, the precise mechanisms and cellular pathways involved this process require further investigation.

However, some limitations of the current study should be noted. First, this study involved a small number of patients, and large-scale multicenter studies are necessary to confirm our findings. Second, we analyzed only a single blood sample for each participant and expression profiling of CLI during disease progression should be measured in the future studies. Third, the molecular mechanisms by which plasma hsa-miR-4739 regulates CLI in patients with T2DM were not fully elucidated.

## 5. Conclusions

In conclusion, our study provides novel evidence of differential patterns of circulating plasma miRNAs in T2DM patients with CLI compared to those without. We show for the first time that elevated plasma hsa-miR-4739 levels are independently associated with T2DM and CLI. Thus, miRNA-4739 is implicated as a novel diagnostic marker and a potential therapeutic target for CLI in diabetes.

## Figures and Tables

**Table 1:** General characteristic of the study subjects for validation study.

	CLI	Non - CLI	*P*
Case, n	26	30	--
Age, years	64.54±2.83	61.50 (57.50-69.25)	0.806‡
T2D Duration, years	15.85±1.48	9.00 (8.00-14.25)	*0.003*‡
Women, %	13, (50.00)	15, (50.00)	1.000†
Hyperlipidemia, %	10, (38.46)	10, (33.33)	0.690†
Hypertension, %	15, (57.69)	9, (30.00)	*0.037*†
Smoking, %	15, (57.69)	8, (26.67)	*0.019*†
ABI	0.58±0.04	1.11±0.01	*0.000*
BMI, kg/m^2^	24.20 (23.30-26.23)	25.54±0.53	0.528‡
CAD, %	7, (26.92)	8, (26.67)	0.983†
Statin use, %	5, (19.23)	4, (13.33)	0.549†
Antihypertensive treatment use, %	7, (26.92)	5, (16.67)	0.351†
Metformin use, %	21, (80.77)	23, (76.67)	0.709†
FPG, mmol/L	9.74 (8.82-14.40)	7.22 (6.13-8.90)	*0.001*‡
HbA1c, %	8.95 (7.88-9.63)	6.90 (6.50-9.05)	*0.002*‡
HDL-C, mmol/L	1.08±0.05	1.16 (0.89-1.31)	0.274‡
LDL-C, mmol/L	2.61±0.18	2.64±0.15	0.920
TG, mmol/L	1.50 (1.06-1.88)	1.29 (0.92-1.86)	0.862‡
TC, mmol/L	2.86 (2.59-4.03)	4.31±0.18	*0.012*‡
Apo A, g/L	1.16 (1.02-0.28)	1.14 (1.06-1.30)	0.172‡
Apo B, g/L	0.92±0.04	0.85±0.04	0.286
Cr, *μ*mol/L	77.50 (58.00-107.45)	65.70 (55.75-72.25)	*0.046*‡
BUN, mmol/L	5.61 (4.60-7.60)	6.15±0.41	0.366‡
UA, mmol/L	324.60±13.53	324.29±19.39	0.899
hsCRP, mg/dL	0.36 (0.19-1.37)	0.65 (0.27-2.53)	0.405‡

ABI, Ankle brachial index; BMI, body mass index; CAD, coronary artery disease; FPG, fasting plasma glucose; HbA1c, glycosylated hemoglobin; HDL-C, high density lipoprotein cholesterol; LDL-C, low density lipoprotein cholesterol; TG, triglyceride; TC, total cholesterol; Apo A, Apolipoprotein A; Apo B, Apolipoprotein B; Cr, serum creatinine; BUN, blood urea nitrogen; UA, uric acid; hsCRP, high-sensitivity C-reactive protein. Data is expressed as mean±SEM, median (interquartile range), or percentage. Significant values are marked in italic. Continuous variables were compared by the Fisher-Pitman Permutation test, and categorical variables were compared by Fisher's exact test in the miRNA array cohort; however, the validation cohort was analyzed using †) *χ*^2^ test or ‡) Mann-Whitney U test, and the others were analyzed using *t*-tests.

**Table 2:** Differentially expressed miRNAs identified by microarray analysis in plasma between T2DM with CLI and without CLI controls.

Differentially expressed type	microRNA name	Mean ratio
Up-regulated	hsa-miR-589-5p	2.0471
	hsa-miR-3132	2.5843
	hsa-miR-4711-3p	2.5770
	hsa-miR-3935	2.6643
	hsa-miR-3168	1.5718
	hsa-miR-4739	3.5206

Down-regulated	hsa-miR-4434	0.4750
	hsa-miR-378i	0.4706
	hsa-miR-3135a	0.2520
	hsa-miR-514a-5p	0.2842
	hsa-miR-378c	0.3074

**Table 3:** Spearman Rho correlations between plasma miRNA-4739 level and CLI risk factors in T2DM patients.

Variable		miRNA-4739
T2D Duration	*rho*	0.113
	*p*	0.408
Hypertension	*rho*	0.094
	*p*	0.492
Smoking	*rho*	0.175
	*p*	0.197
ABI	*rho*	-0.284
	*p*	*0.034*
FPG	*rho*	0.391
	*p*	*0.003*
HbA1c	*rho*	0.297
	*P*	*0.026*
TC	*rho*	-0.127
	*p*	0.350
Cr	*rho*	-0.131
	*p*	0.336

ABI, ankle brachial index; FPG, fasting plasma glucose; HbA1c, glycosylated hemoglobin; TC, total cholesterol; Cr, serum creatinine. All study subjects were included in the analysis. Significant values are marked in italic.

**Table 4:** Binary logistic regression analysis for the risk factors of CLI in T2DM patients.

	**OR**	**95% CI for OR**	***P***	**O** **R** ^**∗**^	**95% CI for ** **O** **R** ^**∗**^	**P** ^**∗**^
T2D Duration	1.189	1.051-1.345	*0.006*	∕	∕	∕
Hypertension	3.182	1.057-9.581	*0.040*	∕	∕	∕
Smoking	3.750	1.220-11.523	*0.021*	∕	∕	∕
FPG	1.377	1.117-1.698	*0.003*	∕	∕	∕
HbA1c	1.730	1.168-2.563	*0.006*	∕	∕	∕
TC	0.550	0.338-0.897	*0.017*	∕	∕	∕
Cr	1.019	1.000-1.038	*0.049*	∕	∕	∕
miRNA-4739	6.610	1.615-27.054	*0.009*	12.818	1.148-143.143	*0.038*

CI, confidence interval; FPG, fasting plasma glucose; HbA1c, glycosylated hemoglobin; TC, total cholesterol; Cr, serum creatinine. ^*∗*^Adjusted for T2D duration, hypertension, smoking, FPG, HbA1c, TC, and Cr. All study subjects were included in the analysis. Significant values are marked in italic.

**Table 5:** Target mRNAs regulated by hsa-miR-4739.

KEGG pathway	Gene Name
Lysosome	HGSNAT, AP1S1, AP1G1, ATP6V0D1, GGA3
Axon guidance	PTK2, RND1, SEMA4G, PLXNA1, NFAT5
Ubiquitin mediated proteolysis	UBE2O, UBE3A, KLHL9, HERC3, UBE2H

## Data Availability

The data used to support the findings of this study are available from the corresponding author upon request.
